# Parathyroidectomy in the treatment of uremic tumoral calcinosis: a 9-year case report and meta-analysis

**DOI:** 10.1186/s12882-025-04507-3

**Published:** 2025-12-06

**Authors:** Haiting Huang, Jun Lu, Jingtian Lin, Peng Huang, Jing Ma, Tingman Huang, Honglian Qin, Yuyi Mo, Jie Wang, Chao Qin

**Affiliations:** 1 The Affiliated Hospital of Youjiang Medical University for Nationalities, Baise, China; 2 Youjiang Medical University for Nationalities, Baise, China; 3 The First Affiliated Hospital of Guangxi Medical University, Nanning, China; 4 Wuming Hospital of Guangxi Medical University, Nanning, China; 5 The Second Affiliated Hospital of Guangxi Medical University, Nanning, China; 6 Key Laboratory of Medical Research Basic Guarantee for Immune-Related Diseases Research of Guangxi, Baise, China; 7 Department of Nephrology, Affiliated Hospital of Youjiang Medical University for Nationalities, No. 18 Zhongshan Road II, Baise, Guangxi Zhuang Autonomous Region China; 8 Department of Neurology, The First Affiliated Hospital of Guangxi Medical University, Nanning, Guangxi Zhuang Autonomous Region China

**Keywords:** Parathyroidectomy, Uremic tumoral calcinosis, Dialysis, Calcium-phosphorus metabolism, Phosphorus management, Secondary hyperparathyroidism

## Abstract

**Objective:**

Uremic tumoral calcinosis (UTC) is a relatively rare but challenging complication in dialysis patients, characterized by abnormal calcium salt deposition. Parathyroidectomy (PTX) is a commonly used treatment; however, some patients experience limited postoperative symptom improvement, suggesting potential factors that affect PTX efficacy. This study aims to investigate the mechanisms underlying suboptimal remission of UTC after PTX to optimize treatment strategies and improve patient outcomes.

**Methods:**

We report a case of a maintenance hemodialysis patient whose UTC worsened after PTX. Following discontinuation of active vitamin D and intensified phosphorus management, the patient’s calcium-phosphorus product significantly decreased, accompanied by marked regression of calcified lesions. To further explore possible mechanisms, we reviewed and analyzed previous literature on PTX treatment for UTC and compared our case with related reports.

**Results:**

Our findings demonstrate considerable individual variability in PTX efficacy for UTC treatment, with calcium-phosphorus product (Ca×P) levels playing a critical role in patient prognosis. This study suggests that calcium-phosphorus metabolism may be a key factor influencing PTX outcomes.

**Conclusions:**

This case highlights the importance of controlling the calcium-phosphorus product in treating UTC.Postoperative management should focus on optimizing calcium-phosphorus metabolism, with particular attention to individualized phosphorus control strategies and appropriate use of active vitamin D.These approaches are crucial for enhancing PTX efficacy and improving long-term patient prognosis.

**Clinical trial number:**

Not applicable.

## Introduction

UTC is a rare but significant complication observed in patients undergoing dialysis, characterized by the deposition of calcium salts in subcutaneous tissues, often resulting in painful and debilitating joint masses [1]. This condition is primarily associated with chronic kidney disease(CKD), where dysregulated calcium and phosphorus metabolism leads to ectopic calcification, particularly in soft tissues such as joints [2]. PTX has emerged as a standard therapeutic intervention aimed at addressing secondary hyperparathyroidism and its associated complications, such as UTC, by reducing parathyroid hormone (PTH) levels and controlling calcium-phosphorus imbalance [3, 4].

Although PTX is widely used to manage UTC, the clinical outcomes following the procedure can be highly variable.While some patients experience significant resolution of lesions, others show limited or no improvement postoperatively [5–8]. This inconsistency in PTX efficacy suggests that multiple underlying factors—such as preoperative calcium-phosphorus balance, the severity of the mineral-bone disorder, and the extent of soft tissue calcification—may influence treatment outcomes. However, despite the common use of PTX in clinical practice, there is limited understanding of the precise mechanisms that contribute to these divergent responses, and few studies have provided comprehensive insights into the factors that may predict or enhance PTX efficacy.

This study addresses this gap by reporting a case of a hemodialysis patient whose UTC worsened after PTX, followed by significant lesion regression through comprehensive management including the rational use of active vitamin D and intensified phosphorus control.To further investigate the factors influencing PTX outcomes, we conducted a comprehensive review of the existing literature on PTX treatment for UTC. By comparing our case with 43 previously published reports, we aimed to identify potential biomarkers and clinical predictors that could help optimize PTX therapy for UTC patients.

Our findings emphasize the critical role of reducing the calcium-phosphorus product in the remission of UTC after PTX, highlighting the significant impact of the rational use of active vitamin D and intensified phosphorus control strategies on improving treatment outcomes.This study provides valuable insights into the pathophysiology of UTC and aims to offer clinicians practical guidelines for improving PTX treatment efficacy, ultimately enhancing patient care and outcomes in UTC management.

## Case presentation

**Fig. 1:**
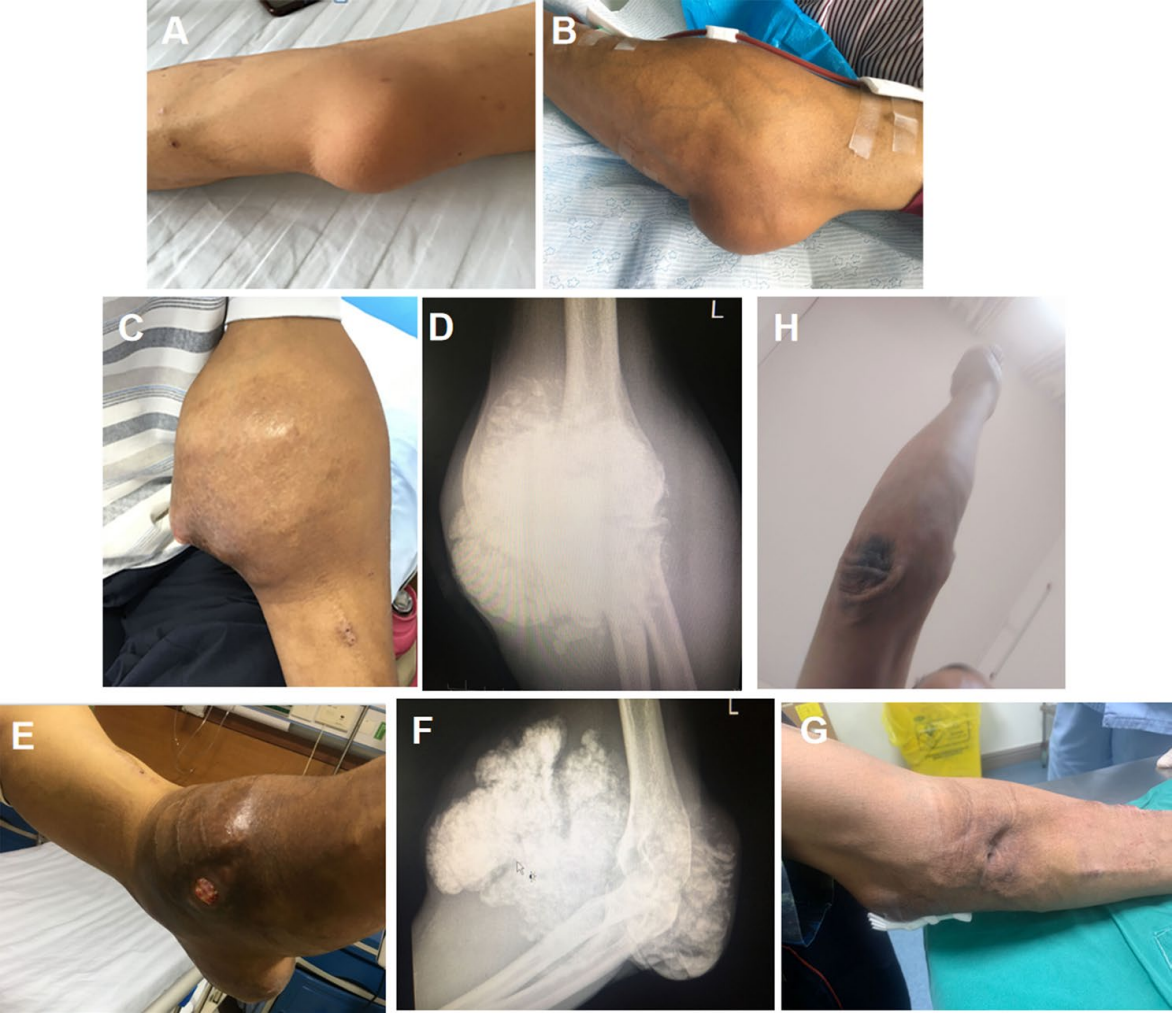
Evolution and Management of a Left Elbow Mass in a Uremic Patient. **(A)** Photograph from 2016 showing an initial nodular mass on the left elbow joint, with intact skin and no signs of inflammation. **(B)** By 2017, the mass had significantly enlarged. **C, D)** Gross appearance and X-ray images of the left elbow mass in 2018. **E, F)** Gross appearance and X-ray images of the left elbow mass in 2021. **G)** By March 2024 (two months after intensified phosphorus-lowering treatment), significant resorption of the mass was observed, accompanied by complete healing of the ulcer and sinus tract. **H)** As of June 2, 2025, the UTC lesion in the patient’s left elbow had been completely resorbed

A 51-year-old male, with no significant medical history—specifically, no history of smoking, alcohol use, hypertension, diabetes, or autoimmune disorders—was diagnosed with uremia in 2014. The patient subsequently commenced a hemodialysis regimen of twice-weekly sessions. In May 2016, a firm, well-demarcated, non-mobile mass was detected on the patient’s left elbow. Initially the size of an egg, the mass was asymptomatic, with no associated pain or functional impairment of the elbow joint, leading to the decision to defer therapeutic intervention (Fig. 1A). However, the mass progressively enlarged, eventually causing restricted elbow mobility, prompting the patient’s re-admission to our department in May 2017.

Upon physical examination, the mass on the left elbow had substantially increased in size, measuring approximately 10 × 10 × 8 cm. The mass was firm at the base but had a soft, non-tender surface (Fig. 1B). Laboratory investigations revealed an intact parathyroid hormone (iPTH) level of 176.42 pg/mL (chemiluminescence immunoassay; reference range,14–80 pg/mL).Radiological imaging showed popcorn-like calcifications within the soft tissues adjacent to the left elbow joint. Due to the presence of an autologous arteriovenous fistula in the left elbow, surgical resection was contraindicated, owing to the heightened risk of hemorrhage.

**Fig. 2:**
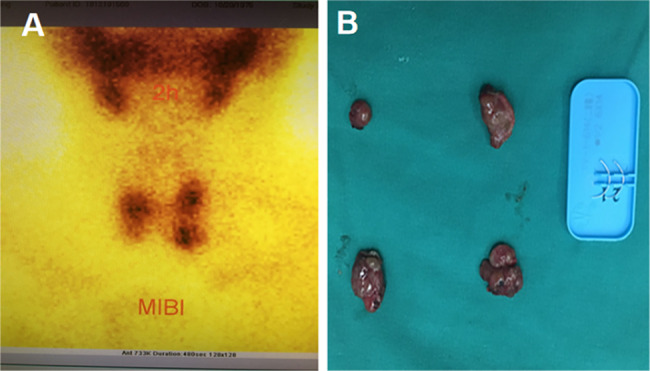
Parathyroid status of the patient. **(A)** Preoperative parathyroid scintigraphy.Shows four hyperfunctioning parathyroid glands, consistent with secondary hyperparathyroidism. **(B)** Intraoperative view of excised parathyroid glands.Displays all four parathyroid glands that were excised

In September 2017, the patient developed a mass on the right shoulder, comparable in size to an adult fist, which progressively enlarged. Upon re-admission in May 2018, physical examination revealed masses measuring 15 × 15 × 10 cm on the left elbow and 9 × 8 × 8 cm on the right shoulder (Figs. 1C and 2A). Digital radiography (DR) of the affected joints exhibited lobulated calcifications (Figs. 1D and 2B). Despite parathyroid scintigraphy ruling out primary hyperparathyroidism, laboratory tests indicated elevated iPTH at 591 pg/mL, along with serum calcium at 2.41 mmol/L and serum phosphorus at 2.5 mmol/L. Based on these findings, the patient was diagnosed with “Chronic Kidney Disease Stage 5,Secondary Hyperparathyroidism, and Multiple Tumor-Like Metastatic Calcifications.”

On May 22, 2018, the patient underwent total parathyroidectomy under general anesthesia, during which all four parathyroid glands were excised (Fig. 2B). Preoperative parathyroid scintigraphy had confirmed hyperfunctioning parathyroid glands consistent with secondary hyperparathyroidism (Fig. 2A). Postoperative histopathology confirmed nodular hyperplasia in all excised glands.

**Fig. 3:**
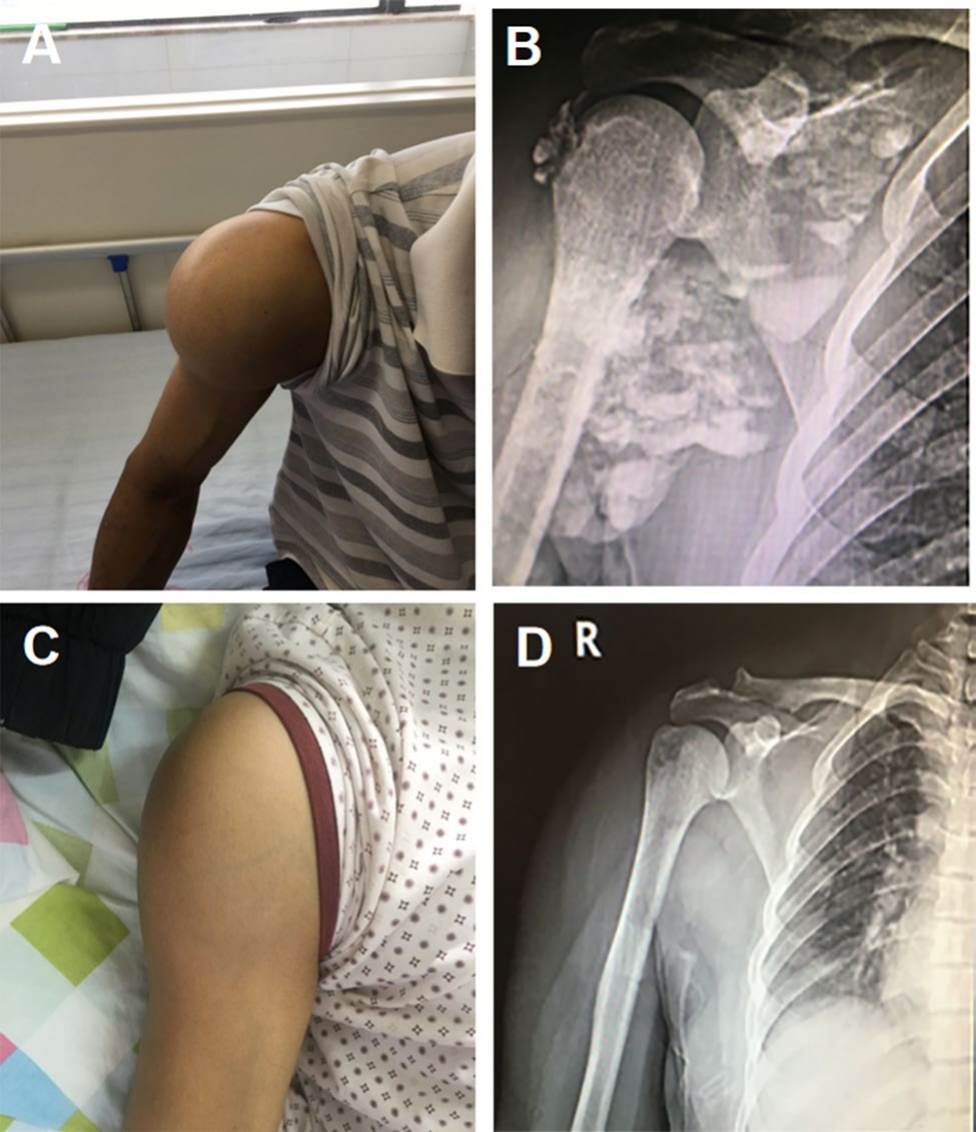
Changes in the Right Shoulder Mass Over Time. **A, B)** Gross appearance and X-ray imaging of the right shoulder mass in 2018. **C, D)** Gross examination and X-ray imaging of the right shoulder five months post-PTX

**Fig. 4:**
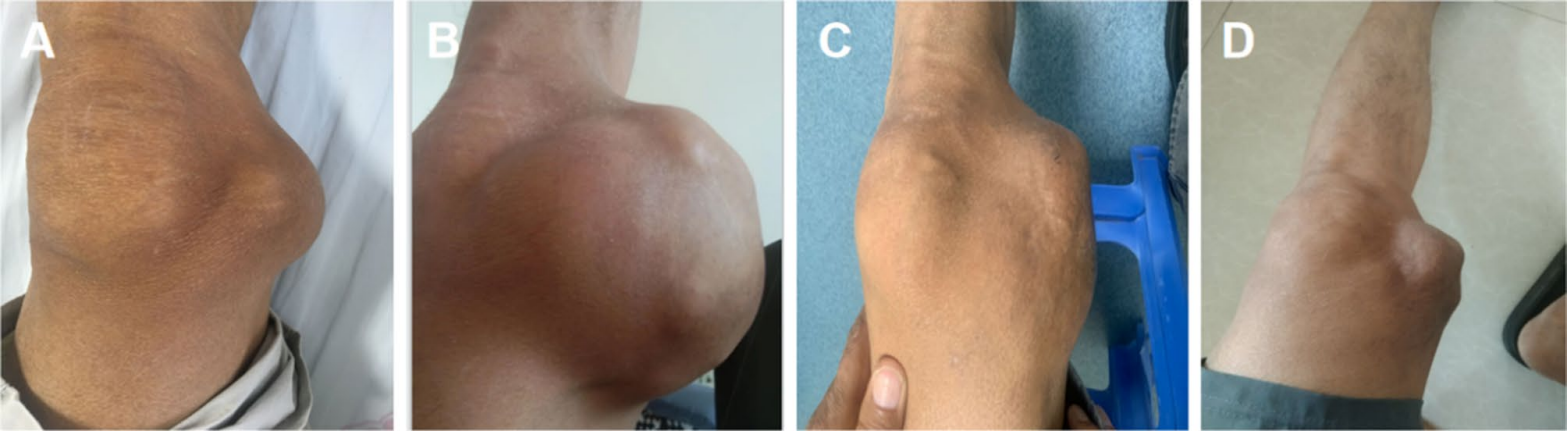
Progression and Treatment Response of a Mass in the Left Knee Joint. **(A)** Initial presentation of the left knee joint mass in March 2021. **(B)** The mass had grown significantly by September 2023. **(C)** In March 2024, notable regression of the mass was observed after 2 months of treatment with sevelamer carbonate. **(D)** By June 2, 2025, the UTC lesion in the left knee joint had further decreased in size

In the five months following the surgery, the right shoulder mass showed notable regression (Fig. 3C and D). However, the left elbow mass continued to enlarge, eventually ulcerating in March 2021. At that time, a new mass was identified on the left knee. Examination of the left elbow revealed a 1 × 1 cm ulcer on the extensor aspect, with abundant discharge of white, sandy granules (Fig. 1E, F). Additionally, a 6 × 6 × 5 cm irregular mass was noted on the left knee (Fig. 4A). From July 2018 to December 2023, both the elbow and knee masses continued to enlarge(Fig. 4B), with persistent secretion of sandy white material from the ulcer on the elbow.

**Fig. 5:**
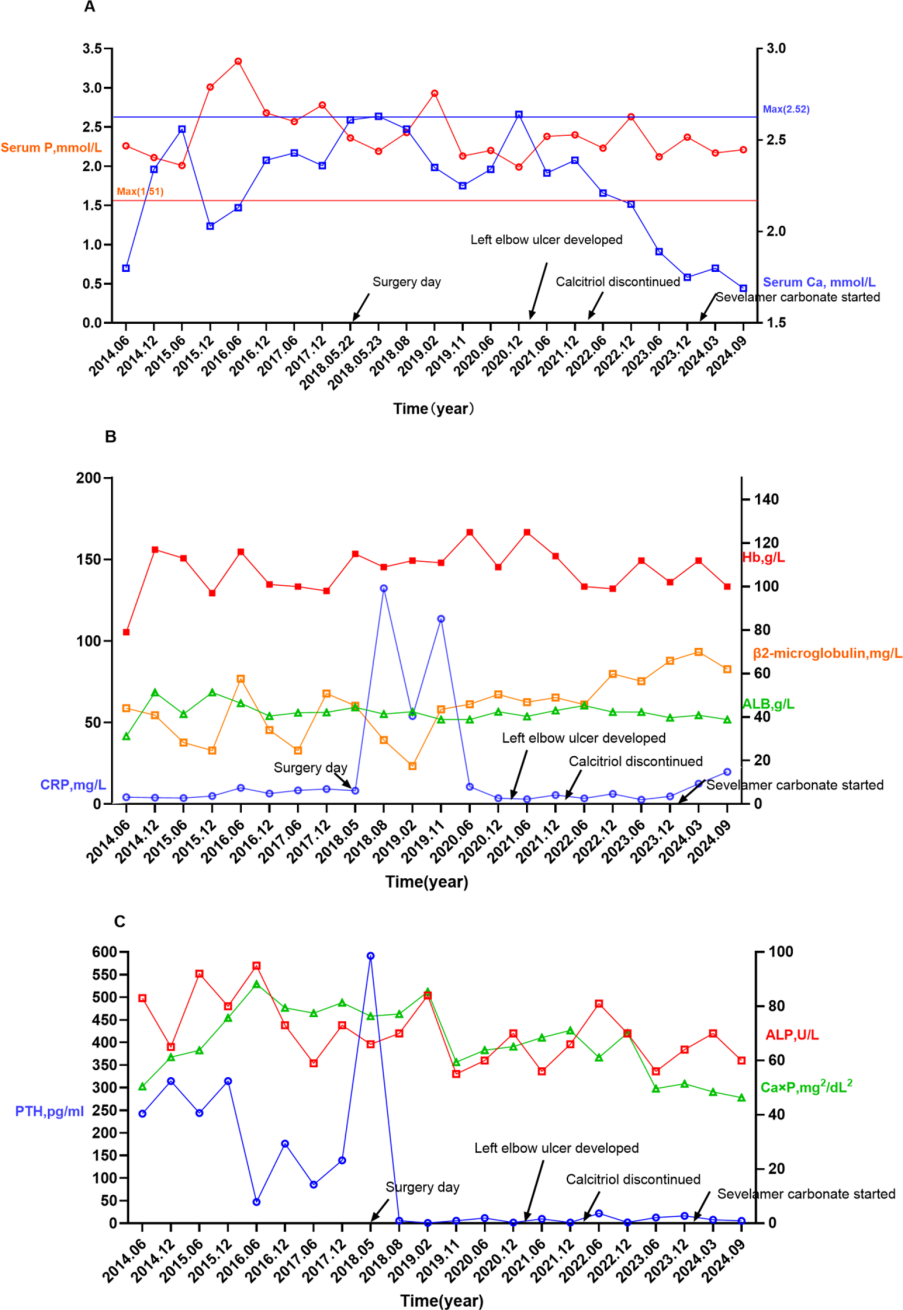
Temporal trends in serum levels of calcium, phosphorus, calcium-phosphorus product (Ca×P), intact parathyroid hormone (iPTH), C-reactive protein (CRP), alkaline phosphatase (ALP), β2-microglobulin, and hemoglobin (Hb) over an 8-year follow-up.Reference ranges: calcium (2.10–2.52 mmol/L), phosphorus (0.85–1.51 mmol/L), Ca × P (< 55 mg²/dL²), iPTH (15–65 pg/mL), CRP (0–6 mg/L), β2-microglobulin (1.0–3.0 mg/L), ALP (45–125 U/L), Hb (130–175 g/L)

In January 2024, the patient was prescribed sevelamer carbonate (1600 mg TID) to reduce phosphate levels. Two months into this treatment, significant regression of the masses on both the elbow and knee was observed (Figs. 1G and 4C). As of June 2, 2025, the UTC lesion on the left elbow had completely resolved(Fig. 1H), and the mass on the right knee had further decreased in size(Fig. 4D).Changes in the patient’s calcium, phosphorus, and iPTH levels over the nine-year follow-up period are illustrated in Fig. 5.The evolution of the patient’s pharmacotherapy and dialysis regimens in relation to the timing of parathyroidectomy is outlined as follows:

### Dialysis regimen and pharmacotherapy

The patient’s dialysis and pharmacotherapy history are summarized in Table 1. Treatment modalities, dialysis frequency, dialysate calcium concentration, and CKD-MBD management were documented in detail across the observation period.

**Table 1:** Timeline of Dialysis regimen and CKD-MBD management

Period	Dialysis modality & access	Frequency & adjunct therapy	spKt/V (StdKt/V)	Dialysate Ca (mmol/L)	CKD-MBD management
Apr 2014 – Jan 2016	Catheter (RIJV)	HD ×2/wk	1.2 (≈ 1.40)	1.5	Intermittent calcitriol 0.25 µg nightly (cumulative 345 µg)
Feb 2016 – May 2018	AVF (L forearm)	HD ×2/wk; HDF ×2/mo; HP ×1/mo	1.1 (≈ 1.33)	1.5	Calcitriol continued
Jun 2018 – Jun 2024	AVF	HD ×3/wk; HDF ×4/mo; HP ×2/mo	1.2 (≈ 2.09)	1.5	PTX performed May 2018; IV Ca for hungry bone prevention; oral calcitriol 0.5 µg nightly ×1 year (165 µg); no Ca carbonate; resumed calcitriol 0.25 µg nightly in 2020–21
2022–2023	AVF, local center	HD regimen not changed	–	1.5	Calcitriol discontinued → gradual Ca decline
Jul 2024 onward	AVF	HD ×2/wk (financial reason); HDF ×4/mo; HP ×2/mo	1.1 (≈ 1.33)	1.5	Sevelamer carbonate 1600 mg TID since Jan 2024

Abbreviations: HD, hemodialysis; HDF, hemodiafiltration; HP, hemoperfusion; AVF, arteriovenous fistula; RIJV, right internal jugular vein; CKD-MBD, chronic kidney disease–mineral and bone disorder; PTX, parathyroidectomy; spKt/V, single-pool Kt/V; Std Kt/V, standardized Kt/V

### Statistical analysis

Continuous variables were presented as mean ± SD for normally distributed data and median (IQR) for non-normally distributed data. Categorical variables were reported as frequencies (percentages). Shapiro-Wilk test assessed normality.For group comparisons, Chi-square or Fisher’s exact test was used for categorical variables. Independent-samples t-test was applied for normally distributed continuous variables, and Mann-Whitney U test for non-normal data.Odds ratios (OR) and 95% confidence intervals (CI) were recalculated using Haldane-Anscombe correction for small sample sizes or zero counts. No multivariate adjustment for potential confounders was performed due to the limited sample size, which is acknowledged as a limitation.Two-sided tests were used with *P* < 0.05 considered significant. Data analysis was done using SPSS 26.

## Results

### Literature analysis

**Fig. 6:**
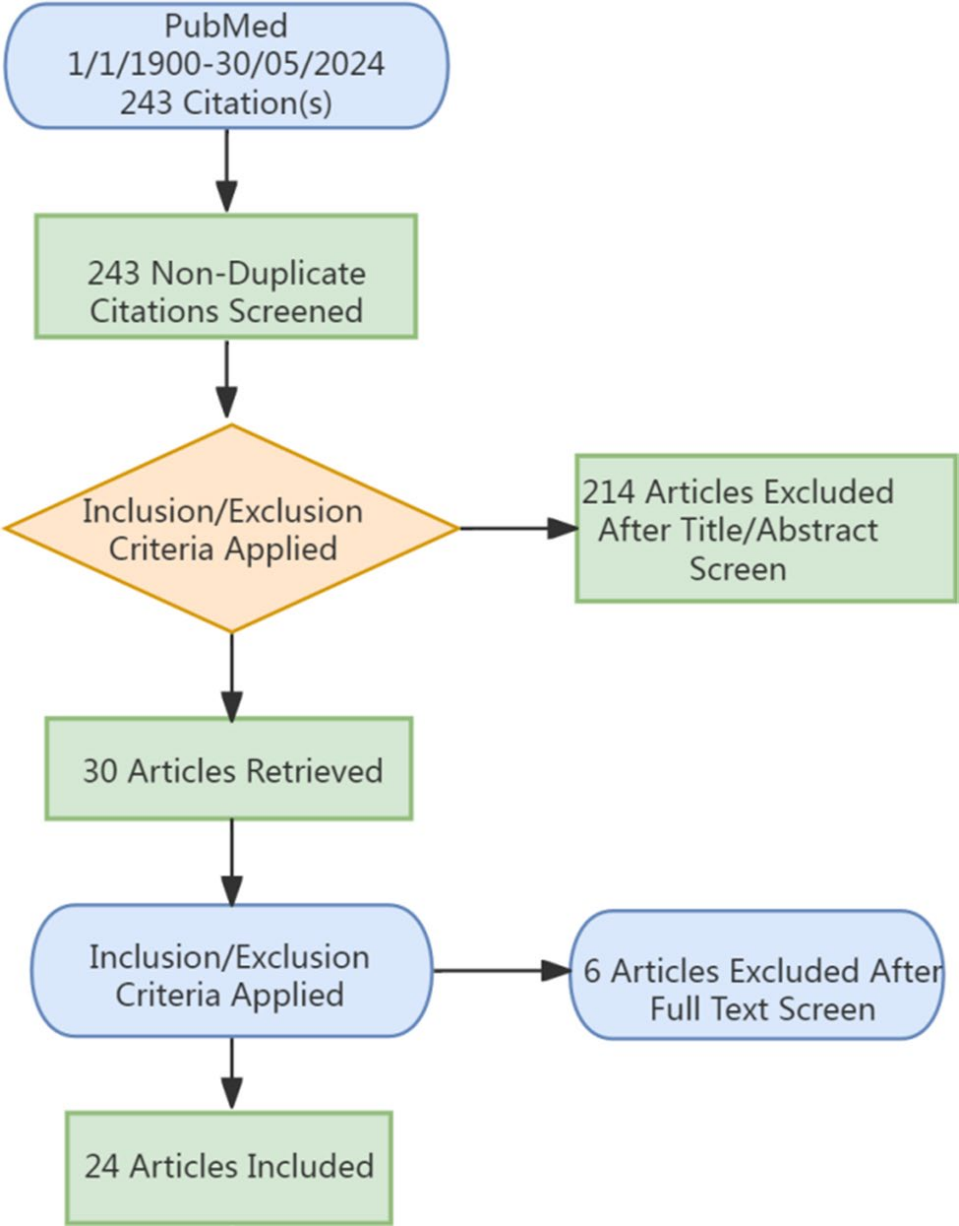
Flowchart of case selection and literature retrieval

An initial search identified 243 articles. After screening titles and abstracts, 214 articles were excluded for not addressing the use of PTX in the treatment of UTC. Five additional articles were excluded due to the unavailability of full text. Ultimately, 24 articles met the inclusion criteria, consisting of 2 case series and22 case reports (including the present case), representing a total of 44 patients (Fig. 6).

### Case report quality assessment

The quality of case reports was assessed by one author, with a secondary random sample quality check performed by another author to validate the initial assessment. The quality of each report was evaluated against the following criteria: gender, age, dialysis vintage, duration of lesion(s), treatment modality, location and number of lesions, and preoperative and postoperative levels of calcium, phosphorus, iPTH, and alkaline phosphatase. Reports missing two or fewer indicators were assigned a “Good” quality rating.Reports missing three indicators were rated “Moderate,” while those missing four or more indicators received a “Poor” rating.

In cases where preoperative and postoperative serum calcium and phosphorus levels were not explicitly reported but could be inferred from context, these data points were considered present for the purposes of quality assessment.

### Quality of literature

The quality assessment of the 44 included case reports varied, with 17 classified as “Good,” 3 as “Moderate,” and 4 as “Poor.” Most case reports (83.3%) provided comprehensive data across critical categories, such as patient demographics (age, gender), dialysis history, lesion duration, iPTH levels, surgical approach, and preoperative and postoperative serum calcium and phosphorus levels. Additionally, information on the number and location of calcific lesions was consistently reported.

### Clinical characteristics of the study cohort

The study cohort consisted of 44 patients, including the present case, with a near-equal male-to-female distribution (20 males, 19 females). The mean age at onset was 38.9 years (range:19–60 years). Notably, the majority of patients (19 out of 21) developed the condition after more than 3 years of dialysis, with a mean dialysis duration of 6.47 years. Only two patients experienced disease onset within the first 2 years of dialysis.

In terms of lesion distribution, 13 patients presented with single lesions, while 30 had multiple lesions.The elbow joint was the most commonly affected site (32%), followed by the shoulder joint (27.2%) and the hip joint (15.9%). Preoperatively, 68.2% of the patients (30 patients) had a calcium-phosphorus product exceeding 55(mg/dL)^2^, and 72.7% (32 patients) exhibited iPTH levels more than eight times the upper limit of normal.

Regarding surgical intervention,54.5% (24 patients) underwent total or subtotal parathyroidectomy. Of these,43.2% (19 patients) received additional forearm autograft transplantation following parathyroidectomy.Postoperative outcomes demonstrated that 75% of patients (33 patients) achieved complete lesion resolution, whereas 25% (11 patients) did not exhibit significant improvement (Table 2).

**Table 2:** Demographic and clinical characteristics of patients (*N* = 44)

Variable	Result
**Age (years)**	38.9 ± 12.21
**Gender**	
Male	20 (44.4%)
Female	19 (43.2%)
Not Mentioned	5 (11.4%)
**Dialysis Duration (years)**	6.47 ± 3.17
**Number of Involved Joints**	
Single	13 (29.5%)
Multiple	30 (68.2%)
Not Mentioned	1 (2.3%)
**UTC Duration (months)**	
≥ 6	24 (54.5%)
< 6	11 (25.0%)
Not Mentioned	9 (20.5%)
**Preoperative Calcium-Phosphorus Product (mg²/dl²)**	
≥ 55	36 (81.8%)
< 55	1 (2.3%)
Not Mentioned	7 (15.9%)
**Preoperative iPTH Levels**	
≥ 8 times normal	32 (72.7%)
< 8 times normal	1 (2.3%)
Not Mentioned	11 (25.0%)
**Postoperative Calcium-Phosphorus Product (mg²/dl²)**	
≥ 55	20 (45.5%)
< 55	21 (47.7%)
Not Mentioned	3 (6.8%)
**Surgical Approach**	
tPTX / sPTX	24 (54.5%)
tPTX + AT	19 (43.2%)
Not Mentioned	1 (2.3%)
**Outcome**	
Resolution	33 (75.0%)
No Resolution	11 (25.0%)

Abbreviations: tPTX, Total Parathyroidectomy; sPTX, Subtotal Parathyroidectomy; AT, Autotransplantation of forearm tissue

### Factors influencing UTC outcomes post-parathyroidectomy

The study cohort included 44 patients, with 33 in the Effective Group and 11 in the Ineffective Group. There were no significant differences between the two groups in terms of age (Effective: 38.21 ± 12.15 years; Ineffective: 42.33 ± 12.98 years; *p* = 0.796), gender (*p* = 0.643), or dialysis duration (Effective: 6.7 ± 3.31 years; Ineffective: 5.5 ± 2.64 years; *p* = 0.302).

Preoperatively, 69.7% of the Effective Group and 90.9% of the Ineffective Group had iPTH levels ≥ 8 times the upper limit of normal, with no significant difference in Ca×P (*p* = 0.750).However, the postoperative calcium-phosphorus product was significantly lower in the Effective Group, with 19 patients achieving values < 55 (mg/dL)^2^, compared to 2 in the Ineffective Group (*p* = 0.021). Additionally, single-joint involvement was significantly more common in the Effective Group (13 patients) compared to the Ineffective Group (0 patients) (*p* = 0.014). The choice of surgical approach was not significantly associated with treatme nt outcomes (*p* = 0.082).

Haldane-Anscombe correction analysis showed a significant association between postoperative calcium-phosphorus product and treatment effectiveness, with an odds ratio (OR) of 6.44 (95% CI: 1.3–31.0), indicating that patients with values < 55 (mg/dL)^2^ were over six times more likely to experience positive outcomes. Joint involvement also showed a notable association, with an OR of 14.54 (95% CI: 0.7–269.7), though the wide confidence interval suggests variability (Table 3).

**Table 3:** Comparison of baseline, biochemical, and clinical characteristics between effective and ineffective PTX groups

Variable	Effective group (*n* = 33)	Ineffective group (*n* = 11)	*P*-value	OR (95%CI)
**Baseline characteristics**				
Age (years)	38.2 ± 12.2	42.3 ± 13.0	0.796	NA
Gender, n (%)			0.643	NA
– Female	14 (50.0%)	5 (45.5%)		
– Male	14 (50.0%)	6 (54.5%)		
Duration of dialysis (years)	6.7 ± 3.3	5.5 ± 2.6	0.302	NA
UTC duration (months)	7.5 ± 5.6	6.2 ± 2.6	0.170	NA
**Biochemical parameters**				
Preoperative iPTH ≥ 8× ULN, n (%)	23 (69.7%)	10 (90.9%)	NA	NA
Preoperative calcium (mg/dL)	9.1 ± 0.8	9.8 ± 1.1	0.450	NA
Preoperative phosphate (mg/dL)	7.5 ± 1.0	7.8 ± 1.2	0.520	NA
Preoperative Ca×*P* ≥ 55 (mg/dL)², n (%)	26 (78.8%)	9 (81.8%)	0.750	NA
Postoperative Ca×*P* ≥ 55 (mg/dL)², n (%)	11 (33.3%)	9 (81.8%)	**0.021**	**6.44 (1.3–31.4)**
Postoperative calcium (mg/dL)	7.8 ± 0.7	9.4 ± 1.0	**0.018**	NA
Postoperative phosphate (mg/dL)	6.2 ± 1.0	7.1 ± 1.2	**0.042**	NA
**Clinical factors**				
Number of involved joints			**0.014**	**14.54 (0.7–269.7)**
– Single	13 (39.4%)	0 (0.0%)		
– Multiple	19 (57.6%)	10 (90.9%)		
Surgical approach			0.082	NA
– tPTX / sPTX	20 (60.6%)	4 (36.4%)		
– tPTX + AT	12 (36.4%)	7 (63.6%)		

Abbreviations: UTC = uremic tumoral calcinosis; PTX = parathyroidectomy; tPTX = total parathyroidectomy; sPTX = subtotal parathyroidectomy; AT = autotransplantation; ULN = upper limit of normal; iPTH = intact parathyroid hormone; Ca×P = calcium–phosphate product; NA = not applicable

## Discussion

Tumoral calcinosis (TC) is a rare soft tissue disorder characterized by periarticular calcium salt deposition. It is primarily classified into three categories: primary non-hyperphosphatemic TC, associated with abnormalities in the SAMD9 gene) [9]; primary hyperphosphatemic TC, stemming from mutations in the FGF-23 gene [10]; and secondary TC, predominantly linked to CKD [11]. In dialysis patients, UTC typically involves large joints, initially presenting as painless masses that may progress to painful swelling and restricted mobility. Diagnosis relies on clinical history, biochemical abnormalities, and characteristic imaging findings.

This study analyzed 44 UTC patients undergoing PTX to explore prognostic factors, with particular attention to calcium-phosphorus metabolism.We also report a nine-year follow-up case in which UTC worsened after PTX but regressed following reduced calcitriol use and intensified phosphorus control, resulting in a significant decrease in Ca×P levels. This observation highlights the pivotal role of postoperative Ca×P management in disease remission and long-term outcomes.

The precise etiology of UTC remains unclear due to limited clinical and basic research [12]. Elevated Ca×P is considered a key factor, with Ca×*P* ≥ 75 (mg/dl)² markedly increasing the risk of soft tissue calcification [13, 14].Current CKD bone metabolism guidelines recommend maintaining Ca×P below 55 (mg/dl)² to reduce calcification risk, though primarily at vascular and non-joint sites [15]. In our review of 44 UTC patients undergoing PTX, 81.8% had preoperative Ca×*P* >55 (mg/dl)², and 81% of patients in the ineffective group maintained elevated Ca×P postoperatively, demonstrating its importance in UTC persistence.While some studies suggest hyperphosphatemia alone is an independent risk factor [16]. our data indicate that both hyperphosphatemia and hypercalcemia may play a role in persistent UTC after PTX.

In this meta-analysis, one patient in the ineffective group developed severe postoperative hypercalcemia [17].Similarly, in our case, hypercalcemia occurred three months after PTX due to excessive calcitriol supplementation, rapidly elevating Ca×P and likely contributing to UTC recurrence. From 2022 to 2024, discontinuation of calcitriol gradually reduced serum calcium and Ca×P levels.When phosphate-lowering therapy (sevelamer) was reintroduced in 2024, even for just two months, the UTC lesion markedly regressed. This clinical course underscores the importance of managing both serum calcium and phosphorus to control Ca×P, not only for disease prevention but also for promoting lesion resolution post-PTX.

Treatment options for UTC include phosphate binders, sodium thiosulfate, low-calcium dialysis, renal transplantation, and PTX, but none have been validated in controlled trials, and treatment failures are common [18–20]. In our cohort of 44 patients, 33 achieved remission after PTX, corresponding to a 75% effectiveness rate. The precise mechanisms underlying UTC remission post-PTX remain unclear, though our findings underscore the significance of calcium-phosphorus metabolism and individualized postoperative management in influencing outcomes.

To identify factors influencing UTC remission post-PTX, we analyzed cases of persistent UTC. In the non-responsive group, 81.8% of patients maintained a post-PTX Ca×*P* >55 (mg/dl)², compared to 36.6% in the responsive group. One illustrative case showed that delayed adjustment of active vitamin D and initiation of phosphate-lowering therapy contributed to persistent UTC [21, 22]. After gradual discontinuation of calcitriol and introduction of regular sevelamer therapy, the patient experienced marked lesion regression within a short period. This clinical course underscores that UTC resolution is closely linked to effective calcium-phosphorus management, highlighting the importance of integrated postoperative metabolic control.

Current evidence suggests a biphasic mechanism underlying UTC remission after PTX.The initial rapid phase is driven by hypocalcemia from hungry bone syndrome (HBS), which occurs in 60–80% of dialysis patients and typically leads to a sharp decline in Ca×P, promoting dissolution of soft tissue hydroxyapatite deposits [23–25]. Many patients experiencing HBS show ≥ 50% reduction in UTC lesion volume within about three months [26, 27].Sustained remission, however, requires long-term maintenance of Ca×P below 55 (mg/dl)² for at least 4–6 months [3, 22].In our case, although no immediate improvement was observed post-PTX, the UTC lesion substantially diminished after reinitiation of sevelamer therapy in 2024, reinforcing the importance of sustained Ca×P control for lasting remission.

In clinical practice, strict management of serum phosphorus is essential for UTC patients with chronic kidney disease. This includes dietary phosphorus restriction, careful use of active vitamin D, and regular monitoring of serum calcium and phosphorus to prevent hypercalcemia and elevated Ca×P, thereby reducing UTC risk and enhancing PTX efficacy. Nevertheless, UTC lesions may regress even when Ca×P exceeds 55 (mg/dl)² post-PTX, indicating that additional, yet unidentified factors may influence treatment outcomes and warrant further investigation.

While secondary hyperparathyroidism (SHPT) is recognized as a risk factor for UTC development [28], the precise role of PTH in UTC pathogenesis remains unclear. Elevated PTH levels do not consistently correlate with UTC incidence but may relate to the extent of remission following PTX [29, 30]. A review of the literature shows that while SHPT contributes to some UTC cases, many patients do not exhibit SHPT. Notably, preoperative PTH levels were significantly higher in the responsive group than in the non-responsive group, indicating that low preoperative PTH may predict non-remission post-PTX. This phenomenon may be related to long-term excessive vitamin D3 supplementation, which can suppress PTH secretion and increase the risk of adynamic bone disease [31].

Bone alkaline phosphatase (ALP), an indicator of bone turnover and skeletal tissue remodeling, has been shown to be closely associated with remission rates of UTC following PTX [27]. In this case, the suboptimal remission of UTC after surgery may be related to the absence of a significant elevation in preoperative ALP levels. Low ALP activity suggests reduced bone resorption and limited skeletal utilization of calcium and phosphate, resulting in increased precipitation of these ions and promotion of tumoral calcinosis. Furthermore, patients with adynamic bone disease typically present with lower ALP levels and experience a milder hungry bone syndrome post-PTX, with no significant short-term decline in the Ca×P [32].In this patient, the lack of significant ALP elevation indicates a limited contribution of bone turnover to the calcium-phosphate burden. Combined with clinical observations, the calcium and phosphate load primarily originated from dietary intake and dialysis management.Discontinuation of active vitamin D and avoidance of calcium-containing phosphate binders (such as sevelamer) effectively improved calcium and phosphate metabolism, promoting lesion regression.However, given the limited sample size and incomplete ALP data, further studies are warranted to clarify the relationship between ALP levels and the efficacy of PTX in treating UTC.

Our analysis also identified joint involvement as a key factor affecting PTX outcomes. Patients with single-joint involvement had significantly better remission rates than those with multiple joints affected, indicating that the extent of calcinosis influences treatment efficacy. This underscores the significance of early intervention in UTC to prevent progression to multi-joint disease, which can complicate management and reduce the likelihood of remission.Some studies suggest that prolonged UTC duration may promote hydroxyapatite crystal formation, which becomes encased in connective tissue and is difficult to absorb [27]. However, our analysis found no significant correlation between disease duration and postoperative lesion regression, highlighting the complex pathogenesis of UTC and the influence of multiple interacting factors.

Our findings emphasize that controlling Ca×P, rather than phosphate alone, is crucial for improving PTX outcomes in UTC. Maintaining Ca×P below 55 (mg/dl)² through dietary phosphorus restriction, phosphate binders, and cautious use of active vitamin D should be a central goal of postoperative management. Early diagnosis and intervention, especially in patients with single-joint involvement, may enhance remission rates and limit disease progression. Individualized strategies targeting calcium-phosphorus balance are essential for optimizing long-term outcomes.

### Limitations

This study has several limitations. First, the small sample size and retrospective design may introduce selection and information biases, limiting the generalizability of our findings. Second, the literature review is primarily based on case reports, which may introduce heterogeneity and reporting bias, further restricting the strength of conclusions. Third, variability in preoperative management and the lack of standardized diagnostic and treatment protocols across centers may have contributed to outcome heterogeneity. Fourth, bone turnover markers such as TRACP-5b and osteoprotegerin (OPG) were not assessed, limiting our ability to fully elucidate the role of bone metabolism in UTC remission after PTX.

These limitations highlight the need for large-scale, prospective, multicenter studies to validate our findings. Future research should standardize treatment protocols, incorporate comprehensive bone metabolism assessments, and develop individualized postoperative strategies targeting calcium-phosphorus balance and bone turnover. Such efforts are essential for improving understanding of UTC pathogenesis and optimizing therapeutic approaches.

## Data Availability

All data generated or analyzed during this study are included in this published article. Further inquiries can be directed to the corresponding author.
